# Identification of gray leaf spot–resistant donor lines in tropical maize germplasm and their agronomic performance under artificial inoculation

**DOI:** 10.3389/fpls.2025.1536981

**Published:** 2025-03-31

**Authors:** L. M. Suresh, Manje Gowda, Yoseph Beyene, Dan Makumbi, Kulai Amadu Manigben, Juan Burgueño, Robert Okayo, Vincent W. Woyengo, Boddupalli M. Prasanna

**Affiliations:** ^1^ Global Maize Program, International Maize and Wheat Improvement Center (CIMMYT), Nairobi, Kenya; ^2^ West Africa Centre for Crop Improvement (WACCI), University of Ghana, Accra, Ghana; ^3^ Maize Improvement Program, Council for Scientific and Industrial Research (CSIR)-Savanna Agricultural Research Institute, Nyankpala, Ghana; ^4^ Biometrics and Statistics Unit, International Maize and Wheat Improvement Center (CIMMYT), Texcoco, Edo de México, Mexico; ^5^ Kenya Agricultural and Livestock Research Organization, Kakamega Research Institute, Kakamega, Kenya

**Keywords:** maize, gray leaf spot, genome-wide association (GWA) study, genomic prediction, DART genotyping by sequencing

## Abstract

Gray leaf spot (GLS) disease is caused by two fungal pathogens, *Cercospora zeae-maydis* and *Cercospora zeina*. The current study evaluated 427 elite tropical/subtropical lines for their responses to GLS under artificial inoculation in Kakamega in western Kenya for 4 years. Furthermore, a subset of 140 lines was used for a high-resolution genome-wide association study (GWAS) for GLS resistance. Among the 427 lines evaluated, 14 were identified as resistant on the basis of a <4 (on a scale of 1–9) GLS disease severity score. Among these 14 lines, three lines, namely CML540, CML559, and CML566, are also known for resistance to MSV, tolerance to drought, and resistance to MLN, respectively. The phenotypic evaluation revealed significant (*P* < 0.01) genotypic and genotype x environment interaction variances and moderate to high heritability for GLS disease severity, area under disease progress curve (AUDPC), and other agronomic traits. GLS disease severity traits were negatively and significantly correlated (*P* < 0.01) with anthesis date, silking date, plant height, and ear height. A subset of 140 lines was genotyped with 33,740 DART-GBS SNP markers. Population structure and principal component analysis grouped the lines into two major clusters with moderate structure in the population. GWAS revealed 13 and 11 SNPs significantly associated with GLS disease severity and AUDPC values. Six among the 13 SNPs detected for GLS resistance are overlapped with earlier studies, which can be used for fine mapping and improvement of GLS resistance through marker-assisted selection. However, SNPs on chromosomes 9 and 10 were unique to the present study. Genomic prediction on GLS traits revealed moderate to high prediction correlations, suggesting its usefulness in the selection of desirable candidates with favorable alleles for GLS resistance. Overall, 14 GLS resistance lines identified in this study can be used as donor lines in both genetic studies and resistance breeding programs.

## Introduction

Maize is the most important cereal crop in sub-Saharan Africa (SSA), growing over 35 million hectares, with an average production of over 70 million metric tons of grain (FAOSTAT, 2021). The crop is mainly grown by several million smallholder farmers for their food security, income, and livelihoods across SSA (Prasanna et al., 2020, 2021). The average maize yield in SSA is very low (∼1.7 tons/hectare) compared to the world average (∼5 tons/hectare) due to various abiotic and biotic stresses (Erenstein et al., 2022). Maize in eastern and southern Africa, specifically in highlands, is affected by many fungal diseases, mainly Turcicum leaf blight (TLB), gray leaf spot (GLS), and Fusarium ear rot (Shi et al., 2007; Kibe et al., 2020a; Nyaga et al., 2020; Omondi et al., 2023; Ndlovu et al., 2024). GLS caused by *Cercospora zea maydis* (Ward et al., 1999) and *C. zeina*, *maydis* (Crous et al., 2006; Liu and Xu, 2013) are major threats to maize production in the world (Ward et al., 1999; Katwal et al., 2013; Omondi et al., 2023);. During the 1960s and ‘70s, the disease was first reported in the USA and later spread worldwide and became a key concern for maize production (Liu et al., 2016) with significant economic yield loss. In SSA, the average yield loss exceeds 70% implicating a significant economic and food security concerns (Vivek et al., 2010; Kinyua et al., 2011; Yigrem and Yohannes, 2019).

Pathogens causing GLS are favored by environmental conditions such as high humidity, moderately high temperature, and extended leaf wetness. Initial symptoms of GLS appear on the lower leaves and are progressively observed in the upper leaves later during the season. The characteristic symptoms of mature GLS lesions are gray to tan in color, sharply rectangular, long and narrow, and run parallel to the leaf veins (Latterell, 1983; Katwal et al., 2013). The GLS damage has been linked to loss of photosynthetic capability and premature plant death (Latterell, 1983; Dodd and McGee, 1989). Due to the widespread impact of the disease in eastern and southern Africa and globally, there is a critical need to adopt effective disease management strategies. The main disease management approaches include the application of fungicides, cultural practices, and, most importantly, host resistance mechanisms (Katwal et al., 2013). Many maize breeding improvement programs in eastern and southern Africa resorted to develop and deploy GLS resistant maize germplasm, as other methods such as the application of fungicides are neither affordable and economical nor environment friendly (Menkir and Ayodele, 2005; Nzuve, 2013; Shi et al., 2014). Hence, breeding for resistant germplasm through conventional methods and integrating advanced molecular tools is the most effective method to control diseases and ensure maize-based food security in SSA.

Finding the resistance source of germplasm and understanding the genetic basis of resistance is critical in managing plant diseases. However, this process is still in progress for GLS resistance. Earlier studies indicated that GLS resistance is a complex trait controlled by multiple genes with small to moderate additive effects (Berger et al., 2014; Benson et al., 2015; Kibe et al., 2020a; Omondi et al., 2023) and strongly influenced by the environment (Clements et al., 2000). Quantitative trait loci (QTL) mapping is an effective tool to understand the genetic basis of complex traits like GLS resistance. Previous QTL mapping studies identified several genomic regions that confer resistance to GLS, which helped to understand its genetic architecture (Zhang et al., 2012, 2017; Berger et al., 2014; Xu et al., 2014; Mammadov et al., 2015; Du et al., 2020; Qiu et al., 2021). QTLs on chromosome bin 5.04 and 5.06–07 were consistently detected in different mapping studies and are of interest to improve GLS resistance (Martins et al., 2019). Meta-QTL analysis revealed that bin 8.08 on chromosome 8 possesses a cluster of QTLs and significant consensus QTLs for GLS, TLB, and southern leaf blight (SLB) with less than 5 cM of confidence interval and also found to be associated with two nucleotide-binding site (NBS) family of R genes (Ali et al., 2013). QTL studies with different populations also revealed QTLs on chromosome bin 8.05/8.06 has been detected for GLS as well as for other foliar diseases like common rust and common smut (Bubeck et al., 1993; Kerns et al., 1999). The consistent genomic regions identified in these studies are needed to focus on improving GLS resistance. However, not many studies were done on eastern and southern African-adapted germplasm.

However, QTL mapping has its limitations, like low mapping resolution due to few recombinations in population development, and each mapping population represents only two alleles. In addition, low relatedness between mapping populations and breeding populations also hampers the translation of the identified QTL into breeding targets. On the contrary, the linkage disequilibrium-based genome-wide association study (GWAS) in a set of mapping panels that represents a broad diversity of the germplasm in breeding programs is a powerful tool for dissecting oligogenic and polygenic traits. GWAS panels with genetically unrelated individuals are expected to accumulate a large number of historical recombination events from the past, which can help to overcome the problems related to the lack of recombination events (Mammadov et al., 2015). In maize, GWAS has been widely used to identify the allelic variants that contribute to improve resistance to many of the maize diseases like maize lethal necrosis (Gowda et al., 2015; Sitonik et al., 2019; Nyaga et al., 2020), GLS (Shi et al., 2014; Kuki et al., 2018; Kibe et al., 2020a; Omondi et al., 2023), sugarcane mosaic virus (Tao et al., 2013), maize streak virus (Nair et al., 2015), common rust (Zheng et al., 2018; Kibe et al., 2020b, Nyaga et al., 2020), Tar spot complex (Mahuku et al., 2016), Fusarium ear rot (Liu et al., 2021), head smut (Wang et al., 2012), and TLB (Poland et al., 2011; Rashid et al., 2020; Ndlovu et al., 2024). Even though several research groups reported GWAS on GLS resistance, the studies on screening a large number of lines to identify the best donor lines and conducting GWAS on lines adapted to SSA are seldom. Therefore, the present research was designed to screen a large number of locally adapted, widely used elite lines and conduct GWAS for GLS resistance in SSA.

GWAS is widely used to find trait-linked markers. The application of trait-linked markers in breeding is limited to large effect QTLs or markers. On the contrary, genomic selection (GS) uses genome-wide markers and captures variations explained by both large-effect and small-effect QTLs or markers, which is effective for complex traits like grain yield and moderately complex traits like GLS and TLB (Kibe et al., 2020a). In GS, the training population has genotypes with both phenotypic and marker data and is used to establish prediction models. From the marker effects estimated from the training population, the genomic-estimated breeding values (GEBVs) are predicted in a testing population that has only marker data but no phenotypic data (Meuwissen et al., 2001). By doing this, we can phenotype only selected lines under controlled environments to produce reliable data. Empirical research has shown the advantage of GS for accelerating the genetic gains per unit of time over phenotypic selection. In maize, GS has been widely applied for many traits for inbred line prediction (Zhao et al., 2012; Technow et al., 2013; Beyene et al., 2019, 2021; Ndlovu et al., 2022, 2024; Sadessa et al., 2022) and hybrid performance prediction (Guo et al., 2019; Schrag et al., 2019; Li et al., 2020). These findings demonstrate the potential of GS to help in the selection of elite lines for disease resistance. The objectives of the present study are (i) to evaluate 427 elite lines for 4 years under artificially inoculated conditions and identify the best GLS resistance donor lines, (ii) to identify the genomic regions associated with GLS resistance through GWAS, and (iii) to examine the potential of GS in predicting GLS resistance and other agronomical traits.

## Materials and methods

**Figure 1:**
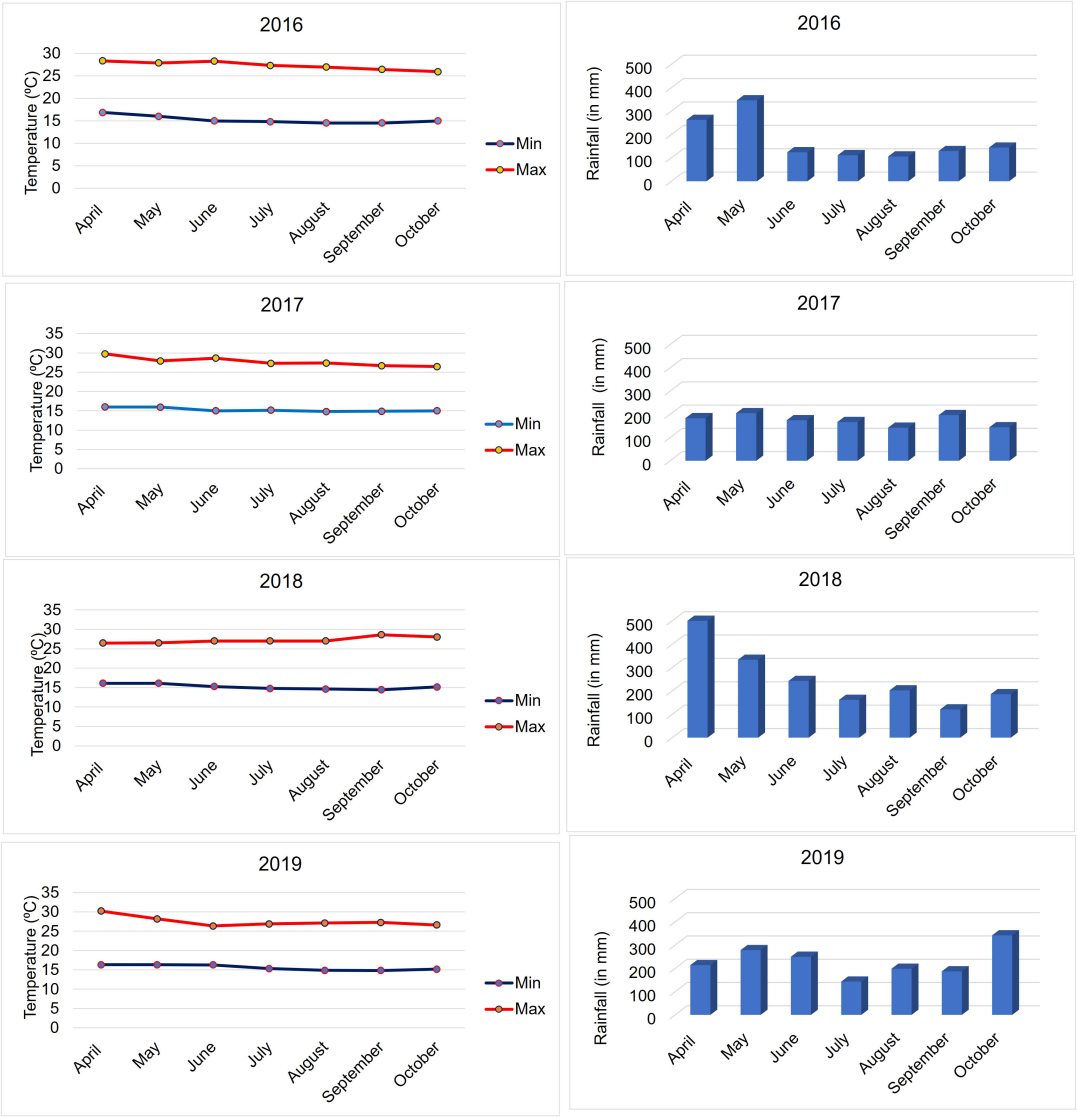
Minimum and maximum temperatures and monthly average rainfall data during the crop seasons at Kakamega for the 4 years (2016–2019) of evaluation.

A collection of 427 elite inbred lines widely used in eastern and southern Africa subtropical maize breeding programs was assembled. These inbred lines have potential resistance sources to various foliar diseases, including GLS. The information on the pedigree and other details is provided in **Supplementary Table S1**. All these lines have been planted to test for their response to GLS under field conditions with artificial inoculation at the KALRO (Kenya Agricultural and Livestock Research Organization) Research Station, Kakamega, Kenya (0°16′N and 34°49′E, 1406 masl) for 4 years (2016, 2017, 2018, and 2019). The monthly average rainfall and temperature (minimum and maximum) in this disease screening period are provided in **Figure 1**. The disease screening location is a hot spot for foliar diseases, including GLS. However, climate change has affected temperature and rainfall patterns in the region, leading to inconsistent disease infection and expression. To have accurate disease severity data, each trial was artificially inoculated to ensure uniform disease expression.

Four hundred and twenty-seven (427) entries were planted in a 5 × 86 alpha (α)–lattice design, randomized and replicated two times each year, by using CIMMYT’s field book (Vivek et al., 2010). These inbred lines were planted in one row of four-meter plots with 15 plants per row. The trial was conducted in the main rainy season (April–September). For these trials, two seeds were planted per hill and later thinned to a single plant per hill three weeks after emergence. This was done to ensure a uniform plant density. All standard agronomic practices were applied during the disease screening period.

### Samples collection for pathogen isolation and inoculation

Leaves from five to ten maize plants with representative GLS lesions were sampled from 20 to 25 fields in western Kenya. The pathogen *Cercospora zeina* was isolated from infected leaves using the single spore isolation method (Meisel et al., 2009; Kinyua et al., 2011). The infected leaves were chopped into smaller pieces (2–5 mm^2^) and surface-sterilized in 5.25% sodium hypochlorite (NaOCl, pure chemical) solution for approximately 30 seconds. The samples were rinsed in sterile distilled water, dried, and plated on potato dextrose agar (PDA) media supplemented with streptomycin sulfate (0.03 g/liter) and incubated at 30°C for five days to allow the pathogen to sporulate in a growth cabinet under a 12h fluorescent light/dark regime. The sporulating fungi were observed under a light microscope, and the hyphal tips of the correct fungi advancing from the colony margins were sub-cultured onto fresh PDA media as part of the culture purification process. A pure culture was stored at −20°C in the KALRO Kakamega Laboratory.

The pathogen from the mother culture was inoculated and multiplied on a susceptible maize host (hybrid PAN4M-19). The conidia were dislodged with a brush and rinsed with 0.01% Tween 20 and the spore concentration was adjusted to a standardized concentration of 4 × 10^4^ spores/ml using a microscope and hemocytometer and applied to all the leaves (V3 stage) of the maize plants with a small brush, and the inoculation was repeated after seven days. During the inoculation period, we walked along the inter-row valley and ensured a uniform inoculum density across the testing population.

### Phenotypic evaluation and data analyses

The inbred lines were evaluated for their responses to GLS in four environments. GLS disease severity is typically at its peak between tasseling and physiological maturity; therefore, disease severity data were recorded at the mid-silking, 77 days after planting (1st scoring called GLS 1) and at the hard dough stage, 105 days after planting (second score called GLS 2). Disease severity was rated plot-wise on the ordinal scale of 1 (highly resistant, without any disease symptoms) to 9 (highly susceptible, with necrosis and completely dead plants). Based on the scoring of 1 to 9 scale disease severity data, we divided the genotypes response as a resistant, moderately resistant, moderately susceptible, and completely susceptible group when the scores were 1 to 4, 4.1 to 5, 5.1 to 7, and 7.1 to 9, respectively. The area under the disease progress curve (AUDPC), a quantitative measure of disease intensity with time, was calculated for each plot to provide a measure of the progression of GLS severity. The AUDPC was computed according to the following equation (Shaner and Finney, 1977):


$$\text{AUDPC}=\sum_{i=1}^{n}[\frac{y_{i}+y_{i+1}}{2}](t_{i+1}-t_{i})$$

where *y_i_* = diseased leaf area estimated on the *i*th disease assessment date, *t_i_* = time (days) from disease onset (i.e., inoculation) to the *i*th disease assessment date, and *n* = total number of disease assessments during the experiment evaluation. Data were also collected for other relevant agronomic traits, namely anthesis date (AD), silking date (SD), anthesis-silking interval (ASI), plant height (PH), and ear height (EH).

GLS disease severity scoring was based on an ordinal scale; therefore, data were checked for conformity with the assumptions of statistical model fitting, that is, normally distributed, constant variance, and independent (Wisniewski and Rawlings, 1990). A plot of residuals against fitted values has shown that the residuals were symmetrically distributed with constant variance for GLS disease severity data and AUDPC values; thus, the data were not transformed. Further data were assessed for homogeneity of variance using Levene’s test before ANOVA, and variances were found to be homogeneous. The phenotypic traits were analyzed, and variance components were estimated with the restricted maximum likelihood (REML) in the ASREML-R (Gilmour et al., 2009) and multi-environment trial analysis (META) R software developed in CIMMYT (Alvarado et al., 2020). The following statistical model was used to estimate variance components:


$$Y_{ijkl}=\;\mu +\;G_{i}+\;E_{j}+\;{\left(GE\right)}_{ij}+\;R{\left(\text{E}\right)}_{kj}+\;B{\left(RE\right)}_{ljk}+e_{ijkl}\;$$

where $Y_{ijkl}$ is the phenotypic observation at the *i*th inbred line, *j*th environment in *k*th replication of the *l*th incomplete block, *μ* is overall means, *Gi* is the genetic effect of the *i*th inbred line, *Ej* is the effect of the *j*th environment, ${\left(GE\right)}_{ij}$ is genotype by environment interaction, $R{\left(E\right)}_{kj}$ is the effect of the *k*th replication at the *j*th environment, $B{\left(RE\right)}_{ljk}$ is the effect of the *l*th incomplete block in the *k*th replication at the *j*th environment, and $e_{ijkl}$ is the residual error. META-R software (Alvarado et al., 2020) was used to obtain best linear unbiased estimates (BLUEs) and best linear unbiased predictions (BLUPs) for all traits. BLUPs were used for GWAS and BLUEs were used for GS analyses. Comparisons of variability between entries were made using the least squared differences (LSD) at a 5% significance level. Broad-sense heritability (H^2^) for the different traits was calculated as the ratio of the estimated genotypic variance to the estimated phenotypic variance (Knapp et al., 1985; Liu et al., 2016).

### Genotypic data analyses

From 427 lines phenotyped, we had genotypic data for 140 lines. Maize leaf tissue samples were collected from 3 to 4 weeks old young, healthy seedlings at the V3 stage. High-quality genomic DNA was isolated from freeze-dried tissues. The Diversity Array Technology (DArT) marker platform was used, and obtained 37,915 single nucleotide polymorphic (SNP) markers. TASSEL ver5.2 (Bradbury et al., 2007) was used to summarize SNP data by site, determine the allele frequencies, and implement quality screening. SNP variants that were monomorphic, called at repeat loci, had a heterozygosity of >0.05 and had a minor allele frequency of <0.05, were filtered, and 19,091 high-quality SNPs were retained for GWAS analysis.

### Population structure and linkage disequilibrium

The population structure of 140 elite lines, which had both phenotypic and genotypic data, was analyzed and sub-grouped using Structure Software 2.3.4 version (Pritchard et al. 2000). The number of discontinuous population structure clusters (K) was predicted from one to five with ten iterations. The true number of population structure clusters (delta K value) was harvested online using an available structure harvester software (Earl and vonHoldt, 2012) based on the highest Ln P (D). The unique population genetic subcluster was represented by each color bar at a *p* = 0.001. The period of length of burn-in was set to 10,000, and Markov Chain Monte Carlo (MCMC) values were set to 100,000 cycles (Evanno et al., 2005).

The kinship matrix was estimated in TASSEL to measure the genetic relatedness among individuals in the association panel. The neighbor-joining tree was developed using the phylogenetic tree analysis in TASSEL software v5.2. Linkage disequilibrium (LD) was calculated using TASSEL software version 5.2. The squared allele frequency correlations (*r* ^2^) between all pairs of SNPs were estimated to determine the extent of LD. The LD decay rate was calculated by using the nonlinear regression model developed by Hill and Weir (1988), with modifications by Remington et al. (2001), was used to fit the LD decay curve into the scatterplot using the LOESS function in R.

### Genome-wide association study

For GWAS, BLUPs across environments were used as phenotypes. Principal components analysis (PCA) was performed using TASSEL ver5.2 (Bradbury et al., 2007). The principal components were used to correct population structure and to create a two-dimensional plot to enable visualization of the probable population structure. A mixed linear model (MLM) that computes both PCs and a kinship matrix (K) was applied for GWAS to correct for population structure (Yu and Buckler, 2006). The R package “FarmCPU—Fixed and random model Circulating Probability Unification” was used for GWAS analysis (Liu et al., 2016). With the GAPIT package, the “hapmap” format of the markers was converted to numeric (0, 1, 2) (Wang and Zhang, 2021). The FarmCPU analysis was performed with a maxLoop of five, where the maxLoop refers to the total number of iterations used. The *p* threshold of 0.01 was used in the model for the first iteration, a quantitative trait nucleotide (QTN) threshold of 0.01 was used in the model from the second iteration, and a minimum MAF threshold of 0.05 was used in the analyses. To determine the significance threshold, multiple testing correction was conducted with the false discovery rate method. The significant associations were declared when *p*-values in independent tests were less than 3 × 10^−4^ (Cui et al., 2016). All the candidate genes for GLS and other agronomic traits located within regions from 5 kb upstream to 5 kb downstream associated with significant QTNs were identified and annotated using the B73 maize reference genome (B73 RefGen_V2) (Schnable et al., 2009; Slaten et al., 2020). The candidate gene annotation information was retrieved from the MaizeGDB database (http://www.maizegdb.org).

### Genomic-wide prediction

Genomic prediction model, ridge-regression BLUP (RR-BLUP), was used to carry out predictions using a fivefold cross-validation (Zhao et al., 2012). BLUEs across environments were used for the analysis. A set of high-quality uniformly distributed 4,983 SNPs with no missing values and MAF > 0.05 was used. We applied a fivefold cross-validation “within population’ approach, where both training and estimation sets were derived from within the association panel. The prediction accuracy was calculated as the correlation between genomic estimated breeding values (GEBVs) and the observed phenotypes. A sampling of the training and validation sets was repeated 100 times for each trait.

## Results

### Weather and disease incidence across the trial years

The amount of rainfall received and ambient temperatures at Kakamega varied during the crop season in all 4 years from 2016 to 2019 (**Figure 1**). The Kakamega region, as expected, consistently experienced high rainfall during the months of April and May, followed by a decline in precipitation during the subsequent months. However, compared to all the other three years, high rainfall and lower temperatures were observed in the months of April and May in 2018. Individual year analyses of experiments revealed significant genotypic variances (**Supplementary Table S2**, **Supplementary Figure S1**) and moderate to high repeatability for all the traits in all 4 years except for GLS1 (77 DAP) in 2017 (**Supplementary Table S2**). The performance of lines against GLS disease severity showed a similar distribution in the years 2016 and 2019, whereas the year 2017 was more divergent (**Supplementary Figure S1**). The correlation between years was positive and significant for GLS disease severity and AUDPC values (**Supplementary Table S3**), which supports combined analyses of the data across years.

### Analysis of variance and heritability

**Figure 2:**
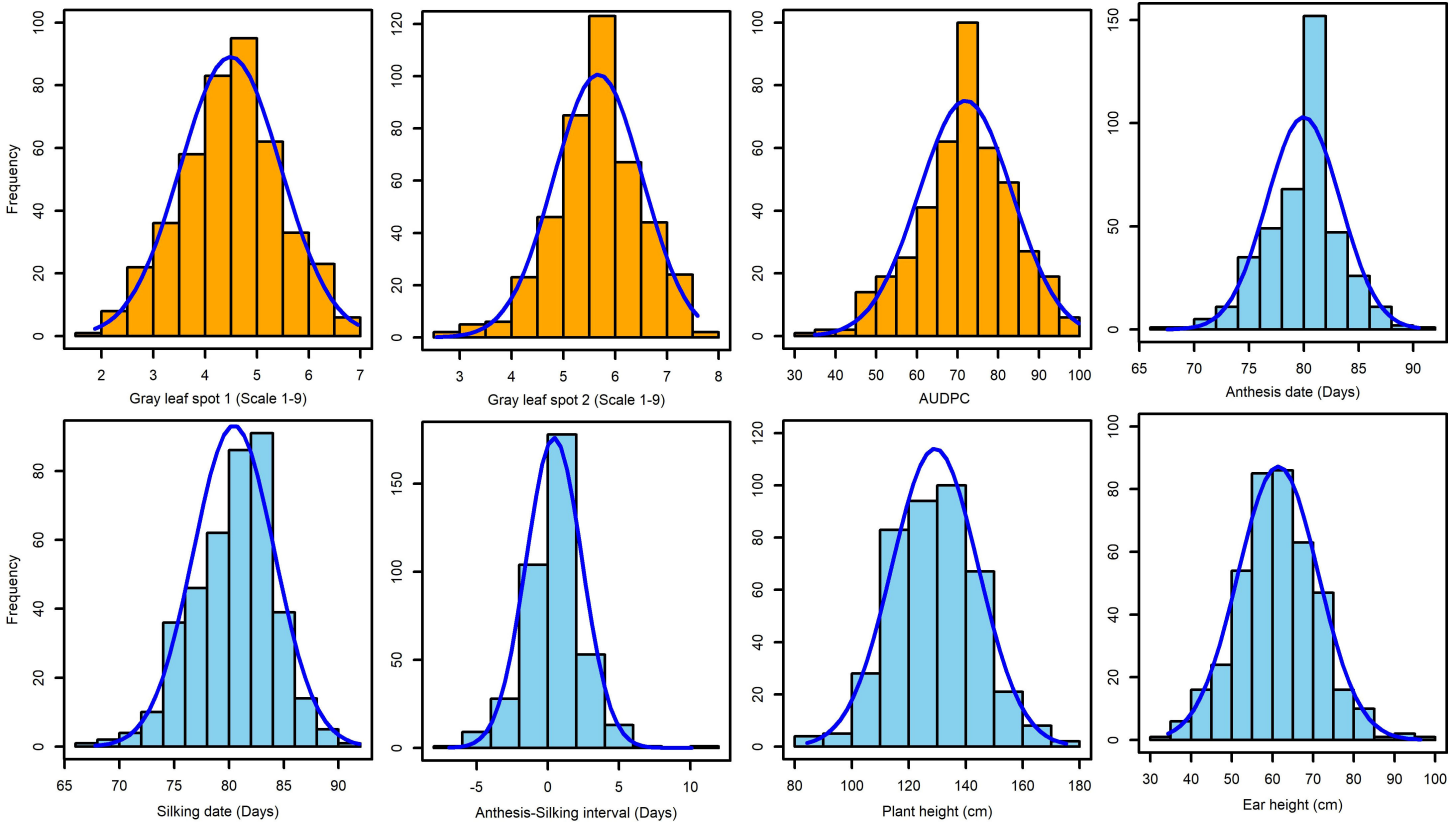
Frequency distribution of GLS disease severity and other agronomic traits. GLS1 and GLS2 = gray leaf spot disease severity data recorded at 77 and 105 days after planting, respectively.

The frequency distribution of GLS disease severity and other agronomic traits showed normal distribution (**Figure 2**). ANOVA across years revealed there were significant variances for genotypic and genotype x environment (GXE) interactions (*P* < 0.05) for GLS disease severity scores and agronomic traits (**Table 1**). The broad-sense heritability estimate for GLS1 (77 DAP) was high (*H* ^2^ = 0.85) compared to the GLS2 (105 DAP) score (*H* ^2^ = 0.57). Broad-sense heritability for agronomic traits was high for AD, SD, and PH, but moderate for EH and ASI. Three susceptible checks, namely CKL150122, CKL150079, and CKL150132 had disease severity scores of >7.3 on a 1–9 scale for GLS2. This high disease severity score on susceptible checks indicates good disease expression in the field (**Table 2**; **Supplementary Table S4**). The first GLS severity score [GLS1 (77 DAP)] varied from 1.9 to 7.0 with a mean of 4.5, while the second score [GLS2 (105 DAP)] varied from 2.5 to 7.6 with an average score of 5.7. The AUDPC ranged from 34.9 to 99.8 with a mean of 71.95. The mean performance of the lines for AD, SD, and ASI was 80, 80.4, and 0.44 days, respectively, and for PH and EH, they were 129.2 and 61.4 cm, respectively (**Table 1**; **Figure 2**).

**Table 1:** Estimation of variance components for GLS disease severity, AUDPC, and agronomic traits evaluated across 4 years (2016–2019) under artificial inoculation.

Genotype	GLS1 (1–9 scale)	GLS2 (1–9 scale)	AUDPC	AD (days)	SD (days)	ASI (days)	PH (cm)	EH (cm)
Mean	4.49	5.67	71.95	79.97	80.44	0.44	129.24	61.40
σ^2G^	1.77**	0.40**	72.03**	8.53**	9.80**	0.88**	110.33**	40.18**
σ^2GxE^	0.40**	0.60**	101.64**	2.26**	2.01**	1.25*	42.81**	31.14**
σ^2e^	1.13	1.25	185.92	8.18	8.61	4.90	278.57	126.35
H^2^	0.85	0.57	0.60	0.84	0.86	0.49	0.71	0.63

*, ** significance at *p* = 0.05 and 0.01 level, respectively. GLS1, GLS2, correspond to disease severity data collected at 77 and 105 days after planting, respectively; AUDPC, area under disease progress curve; AD, anthesis date; SD, silking date; ASI, anthesis silking interval; PH, plant height; EH, ear height.

**Table 2:** Disease severity scores for the best 14 lines and three susceptible checks to GLS and agronomic traits across 4 years (2016–2019).

Genotype	GLS1 (1–9 scale)	GLS2 (1–9 scale)	AUDPC	AD (days)	SD (days)	ASI (days)	PH (cm)	EH (cm)
CKDHL142989	2.5	2.6	34.9	84.0	83.4	−0.8	144.6	76.7
CKL14500	2.1	2.9	35.5	86.8	89.0	2.3	125.9	61.6
CML559	2.1	3.0	38.4	79.7	79.8	0.2	153.4	83.2
CML566	3.0	3.1	48.8	83.5	83.1	−0.2	144.6	67.7
CKL14501	3.2	3.2	45.5	85.0	85.0	−0.1	128.2	63.2
((BRAZIL1546) DH4/CML395)-B-1-2-1	2.8	3.4	49.9	90.5	91.9	1.4	126.8	71.2
CKL155	2.6	3.5	44.6	85.2	84.4	−0.7	145.3	67.8
CKDHL120423	3.0	3.6	43.2	83.0	81.8	−2.2	145.8	73.1
CKLMARS1C3S50196	3.4	3.7	49.4	79.5	81.5	2.1	125.7	61.0
CML540	3.4	3.7	49.7	75.5	75.5	0.1	137.3	49.2
CML536	3.2	3.8	59.9	85.5	85.9	1.0	136.8	61.7
CML574	2.3	3.9	45.5	83.4	82.6	−1.1	138.7	64.9
CKL14529	3.9	4.7	45.3	84.5	82.8	−1.5	142.3	68.1
CML172	4.0	4.8	45.1	74.5	78.0	3.6	118.0	53.1
CKL150079 (Suc. Check)	6.5	7.4	94.8	77.7	79.6	2.0	136.4	63.5
CKL150122 (Suc. Check)	6.2	7.4	90.0	78.2	79.9	2.8	123.7	51.3
CKL150132 (Suc. Check)	5.7	7.4	83.9	78.9	79.0	0.3	131.8	63.0
Mean	4.5	5.7	72.3	79.9	80.4	0.42	129.8	61.9
SE	0.05	0.04	0.56	0.17	0.19	0.10	0.75	0.49
LSD_5%_	1.50	1.29	17.78	4.72	5.02	2.20	20.41	13.42
CV (%)	16.92	11.49	12.52	3.00	3.18	66.17	8.00	11.04

GLS1 and GLS2 correspond to disease severity data collected for GLS at 77 and 105 days after planting, respectively; AUDPC, area under disease progress curve; AD, anthesis date; SD, silking date; ASI, anthesis silking interval; PH, plant height; EH, ear height; SE, standard error; LSD, least significant difference; CV, coefficient of variation.

**Figure 3:**
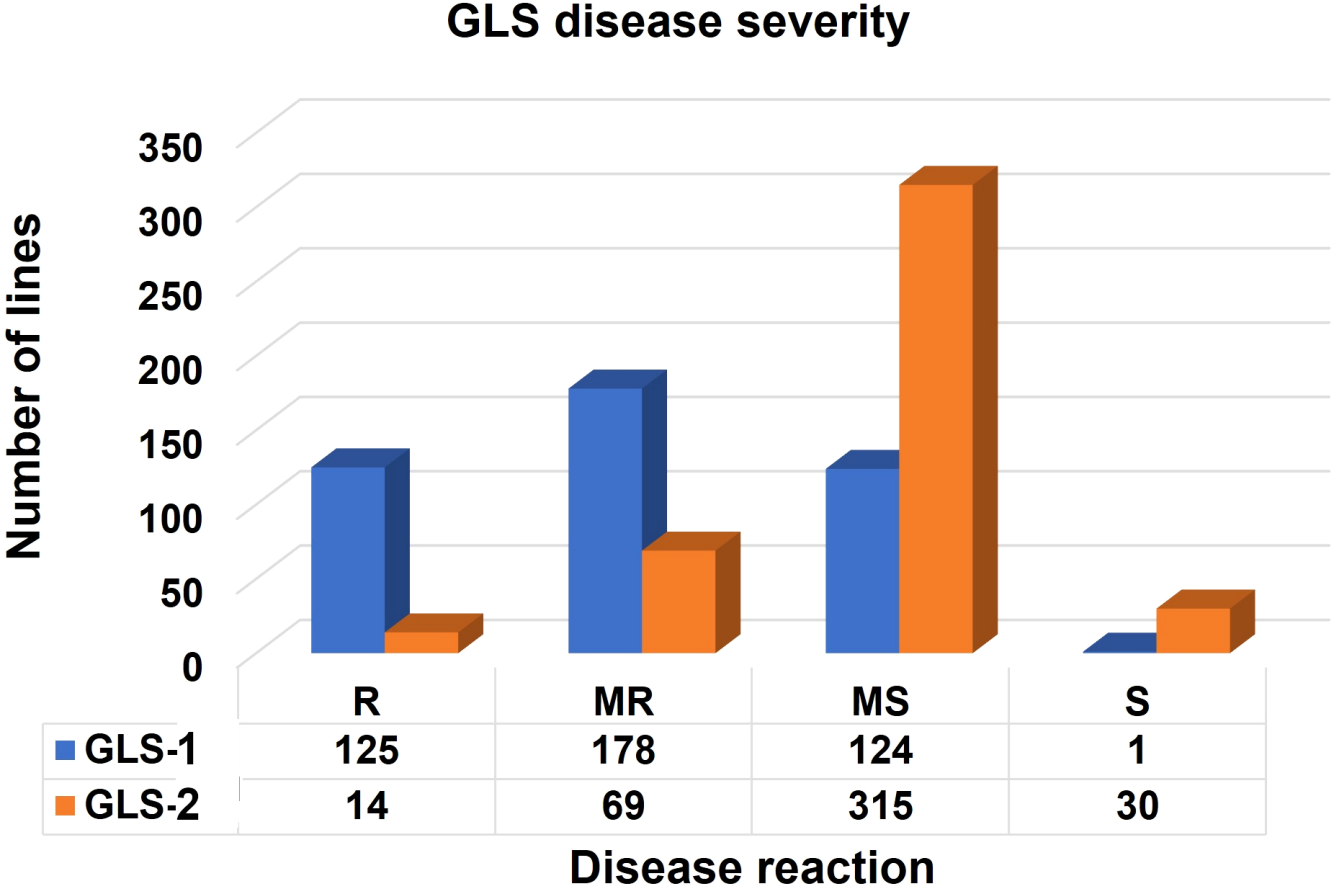
Frequency of the inbred lines with resistant (R), moderately resistant (MR), moderately susceptible (MS), and susceptible (S) reactions to GLS scouted at 77 (GLS1) and 105 (GLS2) days after planting.

**Figure 4:**
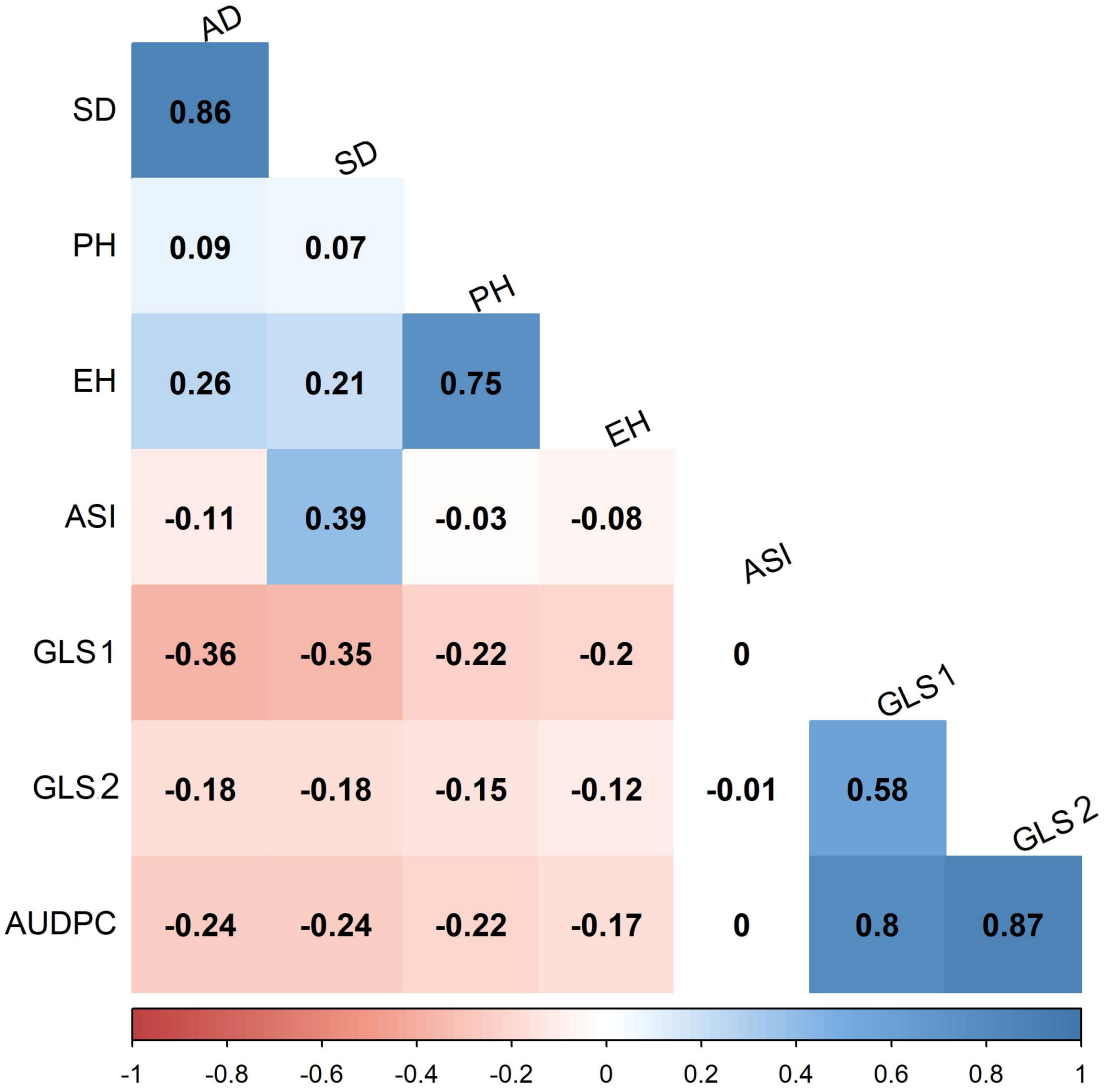
Pearson’s correlation between GLS traits and other agronomic traits evaluated in four environments under artificial infestation of GLS. The correlation level is color-coded according to the color key scale plotted below. Correlations with >0.11 were significant at 0.05 (*p*) level, GLS1, GLS2, correspond to disease severity data collected for GLS at 77 and 105 days after planting, respectively; AUDPC, area under disease progress curve; AD, anthesis date; SD, silking date; ASI, anthesis silking interval; PH, plant height; EH, ear height.

In scoring disease severity on a 1–9 scale, scores with <4 are considered as resistant, 4–5 are moderately resistant, 5–7 are moderately susceptible, and 7–9 as completely susceptible genotypes. Among 427 lines evaluated, 125, 178, 124, and 1 line were resistant, moderately resistant, moderately susceptible, and susceptible, respectively, for GLS1 (77 DAP) disease severity score (**Figure 3**). For GLS2 (105 DAP), 14 lines were resistant while 69, 315, and 30 lines fell into moderately resistant, moderately susceptible, and susceptible categories (**Figure 3**). The 14 GLS-resistant lines also showed a wide range of diversity in their performance for agronomic traits (**Table 2**). The lines CML536 and CKL14501 were not only resistant to GLS but also known to be resistant to TLB (data not shown). Whereas CML566 is also known to be tolerant to drought and CML572 is tolerant to MLN. The results of the correlation analysis between the eight traits including GLS disease severity traits for the maize inbred lines are shown in **Figure 4**. GLS disease severity for both GLS1 (77 DAP) and GLS2 (105 DAP) were significantly and negatively correlated with AD, SD, PH, and EH; however, the magnitude of correlation values was higher at the early stage of the disease severity. AUDPC values were also consistently significant and negatively correlated with AD, SD, PH, and EH. The correlation between GLS traits and ASI was non-significant. The correlation between flowering traits and PH was not significant, whereas EH was positive and significant.

### Marker distribution, population structure, phylogenetic tree, and kinship

**Figure 5:**
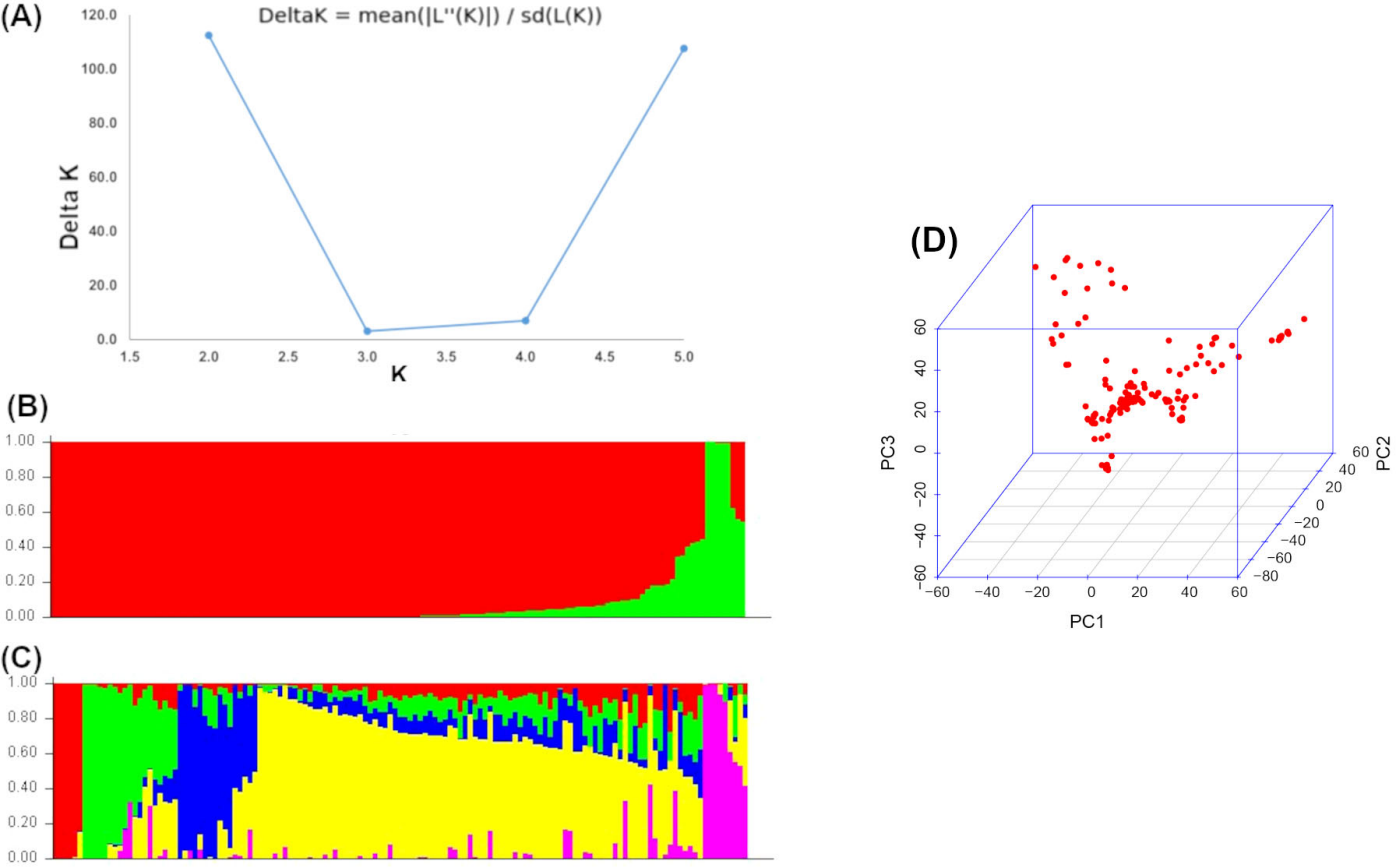
The two and five sub-populations of the 140 inbred lines by using DART SNP markers. **(A)** Best delta K estimation by Evanno method. **(B)** Estimated population structure of tropical maize inbred lines as revealed by DART SNP markers for K = 2 and **(C)** for K = 5. Blue, green, pink, red, and yellow color represents sub-population 1, 2, 3, 4, and 5, respectively. Distribution of inbred lines based on the first three principal components **(D)**.

From 33,740 DART-GBS SNPs used, only 56% (19,091 SNPs) were retained after filtering with the twin criteria of >5% MAF and <10% missing per marker. The number of markers remained ranged from 1,341 on chromosome 10 to 2,876 on chromosome 1. For the final set of markers, the minimum MAF ranged between 0.05 and 0.50. The percentage of missing markers per individual varied from 0 to 10% and the overall average was 4.6%. The proportion of heterozygosity of SNPs (number of taxa that are heterozygous for a given SNP divided by the total number of individuals) ranged from 0 to 0.70, with an overall average of 0.03. The heterozygosity of inbred lines (number of heterozygous markers per inbred line divided by the total number of markers) ranged from 0.008 to 0.27 with an overall average of 0.09. The final set of 19,091 high-quality SNP markers distribution was graphically presented in **Supplementary Figure S2**. The relatedness among the inbred lines used for GWAS analyses was shown with the kinship matrix (**Supplementary Figure S3**). The population structure of 140 diverse maize lines was determined by Bayesian based model in STRUCTURE and PCA (**Figure 5**). The optimum number of K was obtained by plotting the number of clusters (K) against delta K which revealed delta K probability value with two and five clusters based on the highest Ln P(D) values (**Figure 4A**). Evanno table was constructed in the structure harvester with the highest values of 13714.1.45 Ln P(K), 127.46 standard deviations ln P(K), and 107.61 delta K. Delta K value-based line plot had suggested that the population could be structured into two and/or five groups (**Figures 5B, C**). The population structure was also examined by PCA which grouped all lines into two broader groups (**Figure 5D**). An optimal number (K) of three PCs was retained for GWAS.

**Figure 6:**
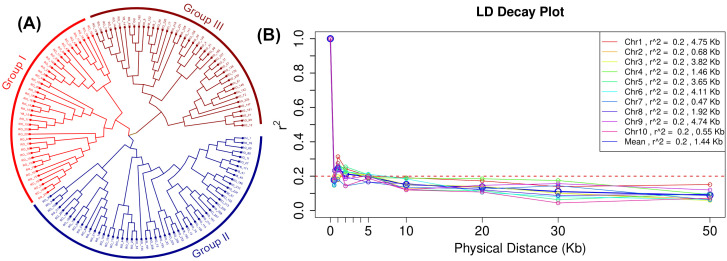
Grouping of 140 inbred lines through phylogenetic tree based on neighbor-joining method **(A)** and linkage disequilibrium (LD) plot **(B)** illustrating the average chromosome-wise and genome-wide LD decay in 140 inbred lines panel using SNPs with call rate 0.9 and minor allele frequency 0.1. The values on the Y-axis represent the squared correlation coefficient r^2^ and the X-axis represents the genetic distance in kilobases (Kb).

The neighbor-joining method-based phylogenetic tree shown in **Figure 6A** revealed that the 140 diverse maize lines can be clustered into three main groups (I = 40, II = 56, and III = 44) differentiated by the different colors (**Figure 6A**, **Supplementary Table S5**). Groups I and II can also be treated as one large cluster with two sub-groups. The genome-wide LD was plotted as LD (*r* ^2^) between adjacent pairs of markers versus the distance between adjacent markers in Kb (**Figure 6B**). The average genome-wide LD-decay in this set of lines is 1.44 Kb at *r* ^2^ = 0.2. LD plots for each chromosome revealed the fastest LD-decay in chromosome 7 (0.47 Kb at *r* ^2^ = 0.2) and chromosome 1 displayed the slowest LD-decay (4.75 Kb at *r* ^2^ = 0.2).

**Figure 7:**
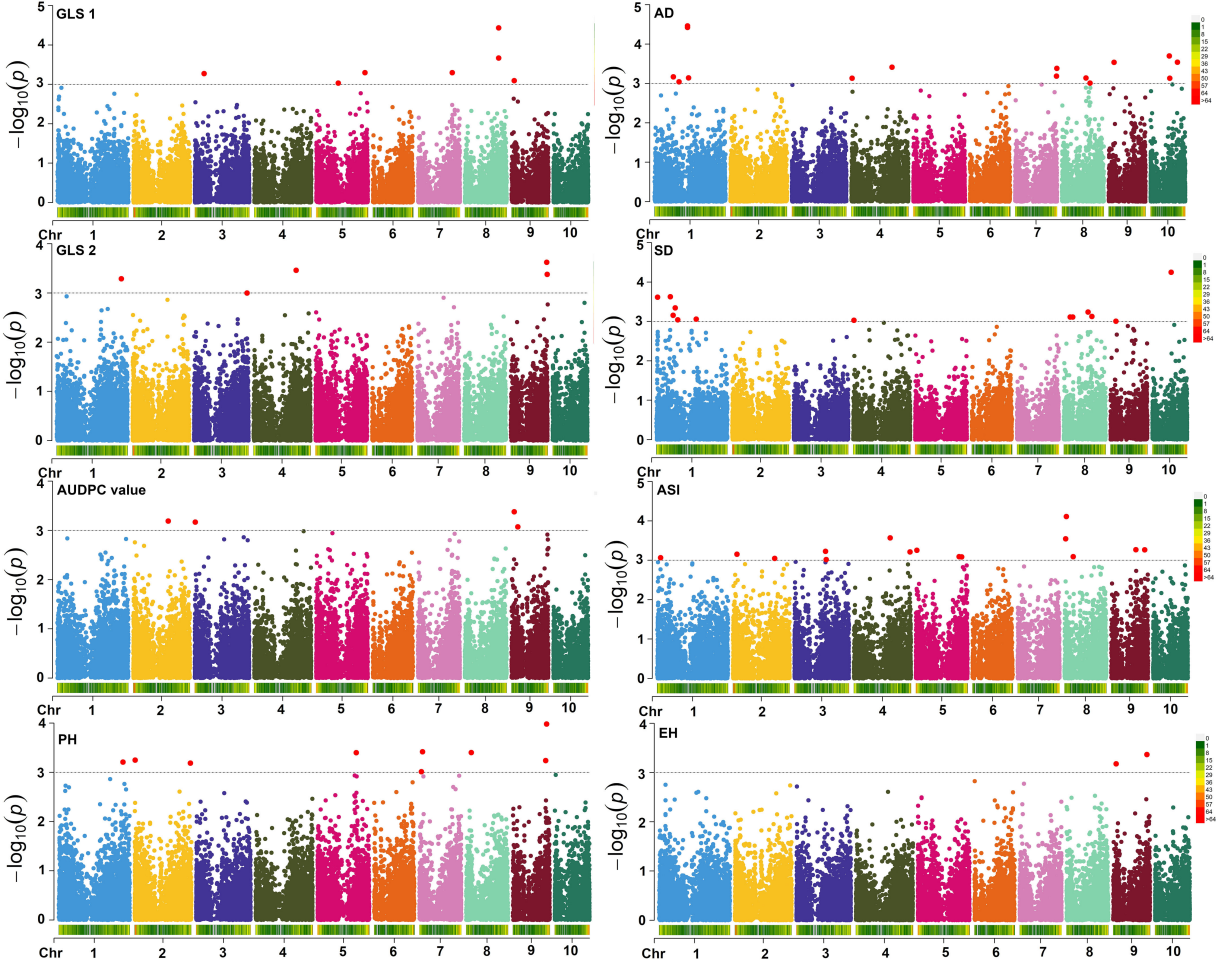
Farm CPU Manhattan plots of GWAS on GLS disease severity and other agronomic traits measured under GLS artificial inoculation. The X-axis shows the SNP position on the chromosome and Y-axis shows the negative log base 10 of the *P*-values; for ease of discrimination, each chromosome was colored differently. The horizontal line portrays the significance threshold (marker *P*-value < 3 × 10^−4^). GLS1, gray leaf spot disease severity data scored 77 days after planting; GLS2, gray leaf spot disease severity data scored 105 days after planting; AUDPC, area under disease progress curve; AD, anthesis date; SD, silking date; ASI, anthesis silking interval; PH, plant height; EH, ear height.

GWAS analyses revealed 69 significant SNPs for eight traits and these results for all traits are summarized using Manhattan plots (**Figure 7**; **Tables 3**, **4**) and QQ plots (**Supplementary Figure S4**). The Q–Q plot of the FarmCPU model showed a sharp deviation from the expected *P* value distribution in the tail area, indicating that false positives and negatives were adequately controlled (**Supplementary Figure S4**). Association analyses for GLS disease severity for GLS1 (77 DAP) revealed nine significant SNPs (**Table 3**). The allelic effect (difference in mean performance for GLS disease severity between inbred lines with major allele and minor allele) for these significant SNPs ranged from 0.45 to 0.39, −0.70 to 0.70, and −3.67 to 5.59 for GLS1 (77 DAP), GLS2 (105 DAP), and AUDPC, respectively under artificial inoculation of GLS. A negative value indicates that the minor allele was the favorable allele associated with increase in GLS resistance by decreasing the GLS severity. For GLS2 (105 DAP), that is, GLS disease severity at a late stage, four significant SNPs were detected. For AUDPC, 11 significant SNPs were identified. The significant SNPs for GLS disease severity were found on all chromosomes with the most significant one being located on chromosome 3 (*p* = 1.37 × 10^−8^). Information on all the significant SNPs, their corresponding MAF, and allelic effects are listed in **Table 3**.

**Table 3:** List of significant SNPs associated with GLS disease severity traits evaluated in four environments under artificial inoculation of *Cercospora zeina*.

SNP	Chr	Position (bp)	Bin name	MLM-P value	MAF	Effect
Gray leaf spot 1						
DT2_153752700	2	153752700	2.06	1.78 × 10^−5^	0.33	−0.24
DT3_18370709	3	18370709	3.04	1.37 × 10^−8^	0.29	−0.35
DT3_129473727	3	129473727	3.05	2.77 × 10^−4^	0.33	0.20
DT4_236484294	4	236484294	4.09	5.49 × 10^−7^	0.08	−0.45
DT5_77120507	5	77120507	5.03	4.39 × 10^−6^	0.39	−0.27
DT9_45478343	9	45478343	9.03	1.67 × 10^−4^	0.24	0.14
DT9_131016039	9	131016039	9.05	9.52 × 10^−6^	0.50	−0.20
DT10_9375453	10	9375453	10.02	1.47 × 10^−5^	0.46	0.20
DT10_124625318	10	124625318	10.04	6.96 × 10^−7^	0.08	0.39
Gray leaf spot 2						
DT3_8260307	3	8260307	3.03	8.30 × 10^−5^	0.10	0.70
DT5_80046482	5	80046482	5.03	2.04 × 10^−4^	0.30	−0.70
DT6_118742764	6	118742764	6.04	1.50 × 10^−4^	0.32	0.40
DT10_7310310	10	7310310	10.02	2.13 × 10^−4^	0.24	−0.45
AUDPC value						
DT1_209722581	1	209722581	1.07	2.18 × 10^−4^	0.36	−2.32
DT1_257413254	1	257413254	1.09	4.11 × 10^−5^	0.12	5.01
DT2_55324276	2	55324276	2.04	1.41 × 10^−4^	0.31	−2.93
DT3_226339092	3	226339092	3.09	8.44 × 10^−7^	0.44	−3.67
DT5_24851058	5	24851058	5.03	1.43 × 10^−5^	0.15	5.59
DT6_1389517	6	1389517	6.00	1.47 × 10^−5^	0.22	−3.46
DT6_91304345	6	91304345	6.02	1.32 × 10^−4^	0.25	−2.81
DT6_165363652	6	165363652	6.08	2.06 × 10^−4^	0.12	3.44
DT7_100370211	7	100370211	7.02	4.39 × 10^−5^	0.42	−2.57
DT8_15011837	8	15011837	8.02	9.07 × 10^−6^	0.23	3.83
DT9_138867161	9	138867161	9.05	1.24 × 10^−5^	0.50	−2.83

MAF, Minor Allele Frequency, effect- Allele Effect, MLM P-value- probability value for the mixed linear model, GLS disease severity, and AUDPC- area under disease progress curve values under artificial inoculation of GLS conditions; Position - The physical position of the SNP (Ref Gen_v3 of B73).

**Table 4:** List of significant SNPs associated with agronomic traits evaluated in four environments under artificial inoculation of *Cercospora zeina*.

SNP	Chr	Position (bp)	Bin name	MLM-P value	MAF	Effect
Anthesis date						
DT3_1407684	3	1407684	3.01	2.03 × 10^−6^	0.34	−0.72
DT3_28537024	3	28537024	3.04	1.25 × 10^−4^	0.05	−1.51
DT3_159134665	3	159134665	3.05	1.33 × 10^−4^	0.06	−1.25
DT3_173798390	3	173798390	3.06	2.39 × 10^−5^	0.35	−0.74
DT5_4115653	5	4115653	5.01	2.18 × 10^−6^	0.15	1.04
DT5_80046482	5	80046482	5.03	5.09 × 10^−6^	0.30	1.21
DT5_190321766	5	190321766	5.05	1.18 × 10^−5^	0.35	−0.70
DT6_114255122	6	114255122	6.04	7.73 × 10^−9^	0.21	1.18
DT6_146530203	6	146530203	6.05	1.68 × 10^−5^	0.30	0.71
DT7_168724486	7	168724486	7.05	2.92 × 10^−8^	0.35	−1.00
DT8_148947397	8	148947397	8.06	1.14 × 10^−8^	0.11	−1.43
DT9_76104516	9	76104516	9.03	1.16 × 10^−10^	0.06	−2.45
DT9_116598435	9	116598435	9.04	1.88 × 10^−4^	0.42	0.64
Silking date						
DT1_36755457	1	36755457	1.03	9.22 × 10^−5^	0.16	−0.76
DT1_160031116	1	160031116	1.05	3.41 × 10^−5^	0.29	−0.76
DT1_196931772	1	196931772	1.06	2.47 × 10^−5^	0.22	−0.84
DT2_215851764	2	215851764	2.08	2.22 × 10^−6^	0.07	−1.31
DT3_1469626	3	1469626	3.01	6.50 × 10^−5^	0.50	0.61
DT3_9577871	3	9577871	3.03	8.45 × 10^−7^	0.44	−0.82
DT3_132334454	3	132334454	3.05	3.16 × 10^−6^	0.07	−1.38
DT4_208901175	4	208901175	4.09	1.29 × 10^−4^	0.06	−1.22
DT5_217466828	5	217466828	5.09	5.50 × 10^−11^	0.16	−1.47
DT6_85907826	6	85907826	6.01	6.26 × 10^−5^	0.06	1.65
DT7_54538519	7	54538519	7.02	2.60 × 10^−5^	0.49	−0.61
DT7_174089319	7	174089319	7.05	1.95 × 10^−4^	0.13	−0.78
DT8_63984569	8	63984569	8.03	3.34 × 10^−5^	0.15	−0.93
DT9_76308336	9	76308336	9.03	4.59 × 10^−6^	0.08	−1.89
Anthesis-Silking interval						
DT5_216421061	5	216421061	5.09	2.09 × 10^−4^	0.21	0.71
DT7_7541489	7	7541489	7.01	3.02 × 10**^−^** ^4^	0.05	1.18
Plant height						
DT2_11667280	2	11667280	2.02	1.28 × 10^−4^	0.14	7.93
DT2_27642609	2	27642609	2.03	1.45 × 10^−4^	0.39	5.16
DT2_27774525	2	27774525	2.03	1.40 × 10^−4^	0.41	5.11
DT6_168920192	6	168920192	6.08	3.31 × 10^−5^	0.06	−12.44
DT8_122249319	8	122249319	8.04	1.69 × 10^−4^	0.09	−8.72
DT8_138595600	8	138595600	8.05	2.14 × 10^−4^	0.18	−6.60
DT10_56267943	10	56267943	10.03	6.73 × 10^−5^	0.05	11.87
DT10_101212899	10	101212899	10.04	8.59 × 10^−5^	0.05	−11.54
DT10_101212965	10	101212965	10.04	7.99 × 10^−5^	0.06	10.78
DT10_101213049	10	101213049	10.04	1.97 × 10^−5^	0.07	10.99
DT10_124620967	10	124620967	10.04	1.99 × 10^−4^	0.10	−8.19
Ear height						
DT5_215513759	5	215513759	5.08	3.32 × 10^−5^	0.12	−5.54
DT7_5660772	7	5660772	7.01	1.61 × 10^−4^	0.14	4.76
DT9_127735255	9	127735255	9.05	6.61 × 10^−5^	0.21	4.21
DT10_101212899	10	101212899	10.04	2.20 × 10^−4^	0.05	−7.02
DT10_101212965	10	101212965	10.04	4.18 × 10^−5^	0.06	7.18
DT10_101213049	10	101213049	10.04	7.69 × 10^−5^	0.07	6.60

MAF, minor allele frequency, effect = allele effect; MLM *P*-value = probability value for the mixed linear model; GLS, disease severity, and AUDPC, area under disease progress curve values under artificial inoculation of GLS conditions; position = the physical position of the SNP (Ref Gen_v3 of B73).

For AD, a total of 13 significant SNPs were detected on chromosomes 3, 5, 6, 7, 8, and 9. One SNP on chromosome 5 (*DT5_80046482*; p = 5.09 × 10^−06^) was common between AD and GLS disease severity at a late stage. The allelic effect for these significant SNPs for AD ranged from −2.45 to 1.21. The largest number of significant SNPs as well as the most significant SNPs in the current GWAS study was identified for SD followed by AD. For SD, 14 significant SNPs distributed across all chromosomes except on chromosome 10 were identified. The SNP on chromosome 5 was the most significant in the current study (*p* = 5.50 × 10^−11^). Two SNPs on chromosome 3 (*DT3_1469626* for SD and *DT3_1407684* for AD) and another two SNPs on chromosome 9 (*DT9_76308336* for SD and *DT9_76104516* for AD) were located closely in the region (**Table 4**). The allelic effect for these significant SNPs for SD ranged from −1.89 to 1.65.

PH and EH are highly correlated agronomic traits in maize. There were 11 significant SNPs associated with PH distributed on chromosomes 2, 6, 8, and 10 whereas there were six significant SNPs detected for EH that were found on chromosomes 5, 7, 9, and 10. Three SNPs on chromosome 10 (*DT10_101212965, DT10_101212899*, and *DT10_101213049*) were common between PH and EH. The allelic effect of significant SNPs for PH ranged from −12.44 to 11.87, for EH, the range varied from −7.02 to 7.18 (**Table 4**).

Candidate genes analysis was conducted for significant QTNs identified in this study. A total of 20 candidate genes were discovered and annotated, among them 3 and 4 candidate genes were identified for GLS1 (77 DAP) and AUDPC values, respectively (**Table 4**). One candidate gene, *Zm00001d015224* is closely associated with both GLS2 (105 DAP) and AD (**Table 5**). Nine candidate genes potentially associated with flowering traits (AD and SD). Similarly, three and one candidate genes were found to be associated with PH and EH, respectively.

**Table 5:** Candidate genes for GLS disease severity traits and other agronomic traits under artificial inoculation of GLS conditions.

Trait	SNP	Chr	Position (bp)	Gene_name	Annotation
GLS1 (77 DAP)	DT4_236484294	4	236484294	Zm00001d053613	RNA-binding (RRM/RBD/RNP motifs) family protein
GLS1 (77 DAP)	DT9_45478343	9	45478343	Zm00001d045883	ADP/ATP carrier protein 1 mitochondrial
GLS1 (77 DAP)	DT10_9375453	10	9375453	Zm00001d023539	protein_coding
GLS2 (105 DAP)	DT5_80046482	5	80046482	Zm00001d015224	Salicylate/benzoate carboxyl methyltransferase
AUDPC	DT5_24851058	5	24851058	Zm00001d013920	Phosphatidylinositol 4-phosphate 5-kinase 9
AUDPC	DT6_1389517	6	1389517	Zm00001d034998	Receptor homology region transmembrane domain- and RING domain-containing protein 2
AUDPC	DT8_15011837	8	15011837	Zm00001d008623	Very-long-chain (3R)-3-hydroxyacyl-CoA dehydratase PASTICCINO 2
AUDPC	DT9_138867161	9	138867161	Zm00001d047673	DUF4378 domain protein
AD	DT3_1407684	3	1407684	Zm00001d039305	RNA polymerase I-associated factor PAF67
AD	DT5_80046482	5	80046482	Zm00001d015224	Salicylate/benzoate carboxyl methyltransferase
AD	DT6_114255122	6	114255122	Zm00001d037159	Os05g0597150 protein
AD	DT9_76104516	9	76104516	Zm00001d046243	Putative ferroportin-domain family protein
SD	DT1_36755457	1	36755457	Zm00001d028486	Chemocyanin
SD	DT1_160031116	1	160031116	Zm00001d030795	Plant calmodulin-binding protein-related
SD	DT3_1469626	3	1469626	Zm00001d039313	protein_coding
SD	DT6_85907826	6	85907826	Zm00001d036370	protein_coding
SD	DT8_63984569	8	63984569	Zm00001d009426	Mov34/MPN/PAD-1 family protein
PH	DT2_11667280	2	11667280	Zm00001d002390	DNA ligase 4
PH	DT2_27642609	2	27642609	Zm00001d002942	Tubulin-folding cofactor B
PH	DT2_27774525	2	27774525	Zm00001d002944	Dolichyl-diphosphooligosaccharide–protein glycosyltransferase 67 kDasubunit
EH	DT7_5660772	7	5660772	Zm00001d018802	protein_coding

GLS1 and GLS2 correspond to disease severity data collected for GLS at 77 and 105 days after planting, respectively; AUDPC, area under disease progress curve; AD, anthesis date; SD, silking date; PH, plant height; EH, ear height.

**Figure 8:**
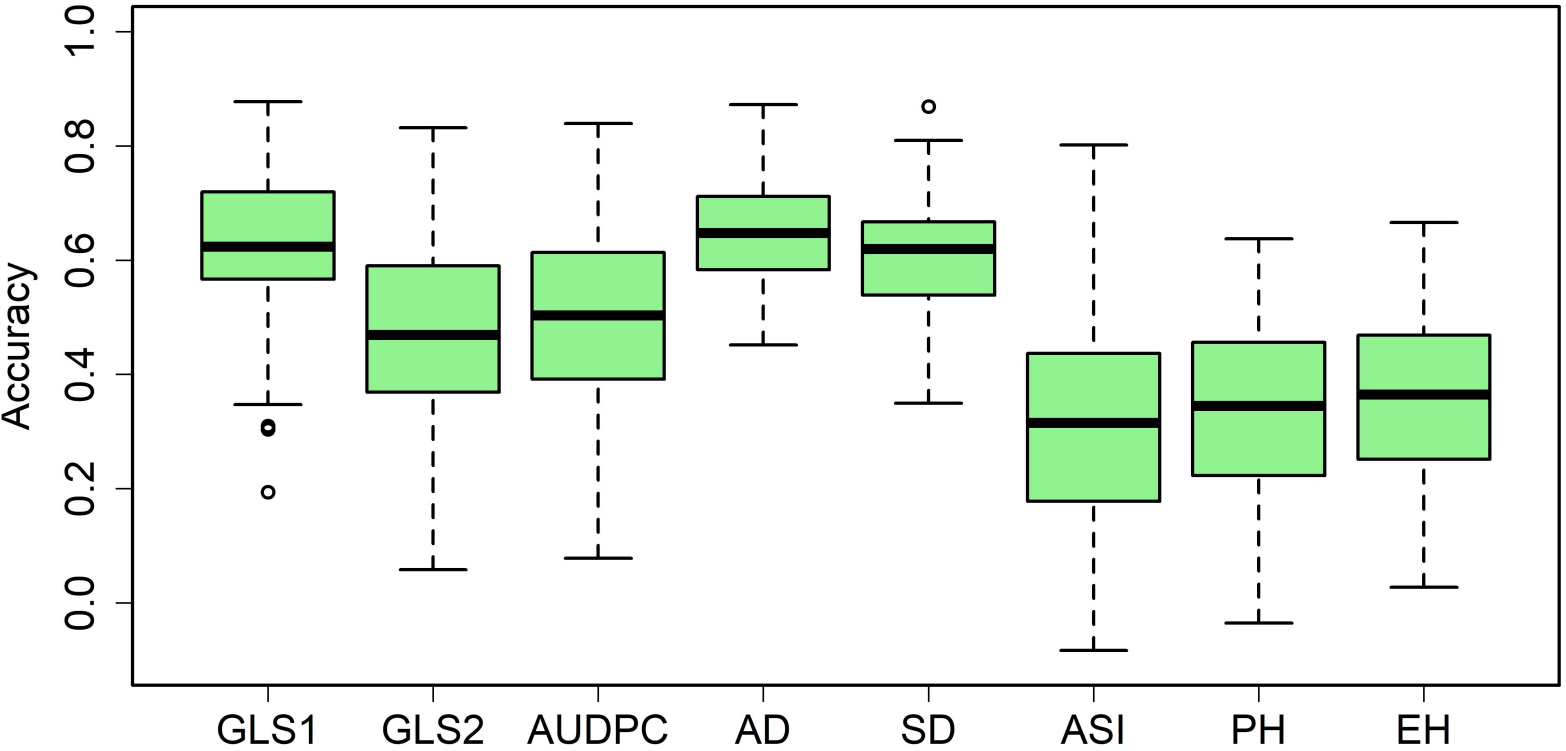
Box-whisker plots for the accuracy of genomic predictions assessed by fivefold cross-validation within the association mapping panel. GLS1 = gray leaf spot disease severity data scored 77 days after planting; GLS2 = gray leaf spot disease severity data scored 105 days after planting; AUDPC, area under the disease progress curve; AD, days to anthesis; SD, days to silking; PH, plant height; EH, ear height.

In most large advanced maize breeding programs, GS is routinely applied. Among the several GS models, G-BLUP and RR-BLUP are computationally less intensive and able to capture both major and minor effect trait variations, so they are well suited for routine application in breeding trials. Therefore, we used the RR-BLUP model to estimate the prediction accuracies in the panel for GLS and other agronomic traits. Prediction accuracies were moderate to high for all eight traits (**Figure 8**). The observed prediction accuracies for GLS1 (77 DAP), GLS2 (105 DAP), AUDPC value, AD, SD, ASI, PH, and EH were 0.62, 0.47, 0.50, 0.65, 0.61, 0.31, 0.34, and 0.36, respectively.

## Discussion

GLS is one of the serious foliar diseases in maize (Kibe et al., 2020a; Omondi et al., 2023) and is caused by *Cercospora zeae-maydis* and *C. zeina*, in Africa *C. zeina* is more prevalent (Crous et al., 2006; Meisel et al., 2009; Liu and Xu, 2013). With the changing climate and increase in cropping the same crop over a larger area, GLS becomes a serious threat to maize production, particularly in smallholder farmers of SSA. To understand the genetics of GLS resistance, in this study, we selected a set of 427 elite lines from tropical and sub-tropical breeding pipelines adapted to eastern and southern Africa and screened them over 4 years in Kakamega under artificial inoculation of *C. zeina*.

Among the 427 lines screened for GLS, 14 were identified as resistant lines with a score of <4 on a 1–9 scale (**Table 2**). These lines represent both the intermediate and late maturity groups, which occupies the major market share in eastern and southern Africa. Among the 14 lines, CKDHL142989 was the best-performing line with a disease severity score of 2.6, followed by CKL14500, which showed a score of 2.9, which is promising to be used as a donor line in GLS resistance breeding. Interestingly, these lines are also tolerant to TLB, which makes them multiple disease-resistant lines. Among the selected 14 lines, CML536, CKDHL120423, CKL14500, CKL14529, and CML540 also showed resistance to TLB (data not shown), suggesting the possibility of using them as donors for resistance to both diseases. CML 536 is also known to be tolerant to drought and low soil nitrogen stress. CML 540 is resistant to MSV, TLB, common rust, early maturing, and tolerance to drought, which provide additional benefits to use these as elite lines in breeding programs. Furthermore, some of these lines may be useful as parents in abiotic stress-tolerant hybrids. For example, line CKL14500, known for GLS resistance, also carries favorable alleles for drought tolerance; it is evident as it derived from two known drought-tolerant lines (CML444 and CML395). The single cross tester (CML444 x CML395) is frequently used as one of the parents for many commercially released drought-tolerant hybrids. Although line CKL14500 has some good trait combinations, it may not be a suitable parent in bi-parental crosses because of its susceptibility to common rust (Sserumaga et al., 2020). Another good line for GLS resistance (CML559) was derived from source germplasm Population 500 (P500), which is resistant to common rust, MSV, and stem borers as well as tolerant to TLB. Another line, CML566 is tolerant to drought with moderate resistance to TLB and MSV. CML574 is a yellow line and is tolerant to MLN, tar spot complex, and fall armyworm and can be used as a donor for multiple diseases and pests. Overall, the identified GLS-resistant lines not only contribute to disease resistance but also carry useful alleles for several economically important traits, including drought tolerance, which makes them multi-trait elite donors.

GLS resistance breeding is influenced by several factors, including genotype, environment, and their interactions. Across environment, analyses revealed significant genotypic and genotype x environment interaction effects for GLS disease severity and AUDPC values (**Table 1**). The magnitude of variance components for genotype x environment interaction variance was approximately 1.5 times of genotypic variance which indicates the role of both additive and non-additive effects. On the contrary, the magnitude of genotypic variance is more than twice that of genotype x environment interaction variance for flowering time traits like AD and SD, and for PH and EH suggesting the major role of additive effects over non-additive effects. A high magnitude of genotype x environment interaction effect was also observed in an earlier study with the IMAS association panel (Kibe et al., 2020a). The heritability estimates for these traits are also on similar expectations with moderate heritability for GLS disease traits and high heritability for flowering traits. Kibe et al. (2020a) also reported moderate heritability for GLS disease severity traits in biparental populations. GLS disease severity and AUDPC values are negatively and significantly correlated with AD, SD, PH, and EH (**Figure 3**). This indicates the selection of lines with early flowering and low to medium height has better resistance to GLS over lines with tall and late flowering plants. These correlations are consistent with earlier studies in association panels evaluated in SSA (Kibe et al., 2020a; Omondi et al., 2023).

GWAS results are influenced by population structure, as it influences marker-trait associations, including the false positives in an association mapping panel. We observed low to moderate population structure with PC1 and PC2 explaining 6.53% and 5.56% of variation, respectively. The association panel lines are broadly grouped into two clusters. This was also supported by phylogenetic tree grouping, though three groups were formed, a closer look shows groups 1 and 2 form one major cluster (**Figure 6A**). Similar findings were also observed by Kibe et al. (2020a) in an association panel and Zhao et al. (2012) in a large set of DH lines which comprised tropical and subtropical lines. Further to understand the structure of the panel used in this study, STRUCTURE software was used, where the *ad hoc* statistics ΔK were used to determine the optimum number of subgroups based on the output log likelihood of data [LnP (D)]. The peaks of the line plot (**Figure 5A**) suggest that the GWAS panel could be broadly divided into two groups or five subgroups. The kinship matrix also suggests moderate structure among the lines used for GWAS (**Supplementary Figure S3**). The moderate structure observed in the panel with no clear differentiation of major adaptation groups is in anticipation of earlier studies where CIMMYT maize germplasm was not found to have a strong population structure (Wu et al., 2016; Rashid et al., 2020). Several researchers have also reported moderate structure in the tropical maize germplasm (Kibe et al., 2020a; Nyaga et al., 2020; Sadessa et al., 2022). CIMMYT’s germplasm pools and populations are known for high genetic diversity and serve as the source of germplasm for many breeding lines in the tropical and subtropical regions (George et al., 2004; Warburton et al., 2005). This is one of the possible reasons we did not observe any well-defined population structure in this study.

The rate of LD decay indicates the presence of diversity in the selected germplasm or panels. Fast LD decay suggests higher diversity at the nucleotide base level, which might have resulted from the historic recombination events. In temperate maize germplasm, LD decay distance (10–100 kb) is several times higher than that of tropical maize germplasm (5–10 kb, Lu et al., 2011). Romay et al. (2013) found that LD decays much more rapidly in the tropical germplasm to about 1 kb at *r* ^2^ = 0.2. A rapid LD decay was observed in each chromosome and across the genome in the panel (1.44 Kb at r^2^ = 0.2). The LD decay observed in this study corroborates earlier studies (Rashid et al., 2020; Kibe et al., 2020a), which suggests the presence of sufficient diversity in the selected set of lines for GWAS.

GWAS revealed 13 markers significantly associated with GLS disease severity and 11 markers with AUDPC value (**Table 3**). Interestingly, though GLS disease severity was positively and significantly correlated with AUDPC values, no common SNPs were detected across GLS traits in GWAS analyses. Nevertheless, there are few regions overlapped in terms of their bin locations (**Table 3**). Many studies reported QTLs for GLS resistance are distributed in all 10 chromosomes, where most of them explained small to moderate effects, except for a very few that had a major effect of >10% and were used for further fine mapping studies (Du et al., 2020; Zhu et al., 2021; Kibe et al., 2020a; Omondi et al., 2023). For three foliar diseases including, GLS, TLB, and SLB revealed 147 multiple disease resistance mQTLs through meta-QTL analyses and identified bins 3.04–08, 5.04–07, and 8.05–06 as significant regions for resistance to these diseases (Ali et al., 2013). Summarizing the earlier QTL studies revealed five major clusters or hot spots for GLS resistance, namely in chromosome 1 at bin’s 1.05–1.06 (Saghai Maroof et al., 1996; Lehmensiek et al., 2001; Balint-Kurti et al., 2008; Pozar et al., 2009; Xu et al., 2014), on chromosome 2 at bin’s 2.03–2.05 (Bubeck et al., 1993; Saghai Maroof et al., 1996; Zwonitzer et al., 2010; Zhang et al., 2012; Lennon et al., 2016), in chromosome 4 at bin’s 4.05–4.08 (Bubeck et al., 1993; Saghai Maroof et al., 1996; Clements et al., 2000; Balint-Kurti et al., 2008; Zwonitzer et al., 2010; Zhang et al., 2012; Benson et al., 2015; Lennon et al., 2016), in chromosome 5 at bin’s 5.03–5.06 (Bubeck et al., 1993; Clements et al., 2000; Lehmensiek et al., 2001; Zhang et al., 2012; Lennon et al., 2016) and in chromosome 7 at bin’s 7.02–7.03 (Bubeck et al., 1993; Pozar et al., 2009; Zwonitzer et al., 2010; Berger et al., 2014; Benson et al., 2015; Mammadov et al., 2015). Comparison of these hot spots with our results revealed two markers on chromosome 2 (*DT2_55324276; DT2_153752700*), three markers on chromosome 5 (*DT5_77120507; DT5_80046482; DT5_24851058*), one marker on chromosome 7 (*DT7_100370211*) were co-located within these regions. This supports the earlier findings and indicates their consistent association with GLS resistance in different genetic backgrounds. These regions are of potential interest to identify possible potential candidate genes and use them to improve GLS resistance.

The other SNPs, though not fallen into these hotspot regions, are overlapped with a few reported QTLs from other studies (Du et al., 2020; Kibe et al., 2020a; Chen et al., 2021; Qiu et al., 2021; Zhu et al., 2021). For instance, the most significant SNP associated with GLS disease severity in this study was *DT3_18370709* at the physical position 18.37 Mb on chromosome 3 (*P* = 1.37 × 10^−8^) was overlapped with “consensus QTL” on bin 3.04 in the IBM2005 map (Shi et al., 2007), and the QTL (*qYCM-DS3-1*) reported in the RIL population (Chen et al., 2021). Another SNP (*DT3_129473727*) at bin 3.04 is overlapped with an earlier reported SNP through GWAS (Kuki et al., 2018) and QTL mapping (Du et al., 2020). All three SNPs detected in this study were also overlapped with an earlier study by (Kibe et al., 2020) in the GWAS panel and biparental populations. The remaining markers, especially on chromosomes 9 and 10, appeared to be specific for the current study and new additional sources for GLS resistance.

Flowering traits, both AD and SD, also play crucial roles in selecting GLS resistance. SNP (*DT5_80046482*) at chromosome 5 is significantly associated with AD and also showed a strong association with GLS disease severity (**Tables 3**, **4**). Further, we also observed eight bins, namely, bins 3.03, 3.04, 3.05, 4.09, 5.03, 6.04, 7.02, and 9.03 shared SNPs for both GLS disease severity and flowering time traits (**Tables 3**, **4**). Other agronomic traits like PH and EH are also significantly and negatively correlated with GLS resistance. A comparison of markers detected for GLS disease severity and PH and EH revealed three bins, bins 6.08, 9.05, and 10.04 shared the markers. These findings suggest strong linkage or clustering of markers in certain regions of the genome and selecting early flowering time, and medium height also indirectly helps in improving GLS resistance.

Among the eight candidate genes identified in this study for GLS resistance, one on chromosome 5 (*Zm00001d015224*) associate with both GLS and AD encodes for Salicylate/benzoate carboxyl methyltransferase (**Table 5**). Salicylate carboxyl methyltransferase responsible for formation of methyl salicylate which plays important role in signaling for local defense and systemic acquired resistance of plants against pathogens invasion (Koo et al., 2007). Another candidate gene associated with AUDPC value on chromosome 5 (*Zm00001d013920*) encodes for Phosphatidylinositol 4-phosphate 5-kinase which plays a role in plant defense and cellular function (Zarreen et al., 2023). Another candidate gene encodes for receptor homology region transmembrane domain- and RING domain-containing protein involved in transport of storage proteins to protein storage vacuoles. Overall, the identified candidate genes in this study are involved in plant defense and development.

GLS resistance-linked QTLs are reported across all chromosomes. Some of the major effects of QTLs on chromosomes 1, 2, 5, and 8 were also fine-mapped (Liu et al., 2016; Zhang et al., 2017; Du et al., 2020; Qiu et al., 2021). As the number of QTL needed to be considered to achieve maximum resistance to GLS increases, it complicates the success of marker-assisted selection. Unlike GWAS, which identifies the markers linked to the trait of interest, GS calculates the estimated breeding values of the trait/s for genotypes in practical breeding which is used to select superior-performing candidates. GS has a high predictive power since it uses genome-wide markers to predict the breeding value of individuals in the testing population (Meuwissen et al., 2001). The rapid innovation in next-generation sequencing technology able to produce millions of markers with reduced cost of genotyping makes GS a critical method in breeding programs. Currently in maize, GS is used for complex traits like grain yield and drought tolerance (Zhao et al., 2012; Beyene et al., 2019, 2021b). Compared to abiotic stress traits and grain yield, diseases like GLS are relatively less complex, following this expectation, we observed a high prediction correlation for GLS disease severity and a moderate correlation for AUDPC values (**Figure 8**). These correlations are higher than observed correlations in IMAS association panel for GLS disease severity but on par with correlations observed in DH populations (Kibe et al., 2020a; Omondi et al., 2023). GS on several disease traits showed promising results with a prediction accuracy of as high as 0.70 for TLB (Technow et al., 2013) 0.86 for MLN (Sitonik et al., 2019), and moderate accuracy of 0.46 for Fusarium ear rot resistance (Holland et al., 2020; Kuki et al., 2020) and common rust resistance (Kibe et al., 2020b; Nyaga, et al., 2020). By using a large, related, and improved training population, the prediction accuracy could be greatly elevated as shown for GLS in the previous study, GS accuracy was low-to-moderate with a range of 0.29–0.56 for GLS resistance with a small training population, which was elevated to 0.77 when increasing the diversity and size of the training set (Kibe et al., 2020). Therefore, complementing GS with phenotypic selection is promising to achieve high genetic gain for GLS resistance with optimal resources.

## Conclusion

In this study, 427 diverse tropical maize inbred lines were evaluated in 4 years under artificial inoculation of GLS (*Cercospora zeina*) in Kakamega, Kenya. Wide variation was observed for GLS disease severity and AUDPC values across years, but significantly and negatively correlated with agronomic traits such as flowering time and plant height. We identified fourteen GLS-resistant lines that can be used as either donors or parents in a resistance hybrid breeding program. In SSA, three-way cross hybrids are the final commercial products. Therefore, using lines with moderate resistance to GLS can combine the desirable alleles from all three parents, which also adds up to higher resistant hybrids. From 427 lines a set of 140 lines were genotyped with DART GBS genotyping. Population structure analyses revealed moderate structure in the panel. GWAS analyses revealed 24 SNPs significantly associated with GLS traits. Most of the detected SNPs were also co-located with earlier studies for GLS resistance. This indicates the consistency in the detection of the markers across genetic backgrounds. GS prediction correlations are moderate to high, which opens new avenues to improve breeding for GLS disease resistance with optimum allocation of resources.

## Data Availability

The datasets presented in this study can be found in online repositories. The names of the repository/repositories and accession number(s) can be found in the article/**Supplementary Material**.
