# Supplementary material for: Identification of gray leaf spot–resistant donor lines in tropical maize germplasm and their agronomic performance under artificial inoculation
Source: Front Plant Sci. 2025 Mar 31;16:1536981. doi: 10.3389/fpls.2025.1536981 (PMC11997715; doi:10.3389/fpls.2025.1536981)
Supplement: Supplementary file 2 [file DataSheet1.docx]

**Genome-wide association and genomic prediction in East African tropical maize germplasm reveal novel and known genomic regions for resistance to gray leaf spot**

**Suresh, L.M^1^., Manje Gowda^1*^, Yoseph Beyene^1^, Dan Makumbi^1^, Manigben Kulai Amadu^1,3,4^, Juan Burgueño^2^, Robert Okayo^1^, Vincent W. Woyengo^5^, and Boddupalli, M. Prasanna^1^**

^1^International Maize and Wheat Improvement Center (CIMMYT), P.O. Box 1041–00621, Nairobi, Kenya.

^2^Biometrics and Statistics Unit, International Maize and Wheat Improvement Center (CIMMYT), El Batán, Texcoco Edo de México, México

^3^ West Africa Centre for Crop Improvement (WACCI), University of Ghana, PMB 30 Legon, Accra, Ghana

^4^ CSIR-Savanna Agricultural Research Institute, PO. Box 52, Tamale, Nyankpala, Ghana

^5^ Kenya Agricultural and Livestock Research Organization, Kakamega Research Institute, P.O. Box 169‒50100, Kakamega, Kenya.

* Correspondence: [m.gowda@cgair.org](mailto:m.gowda@cgair.org)

Supplementary Material


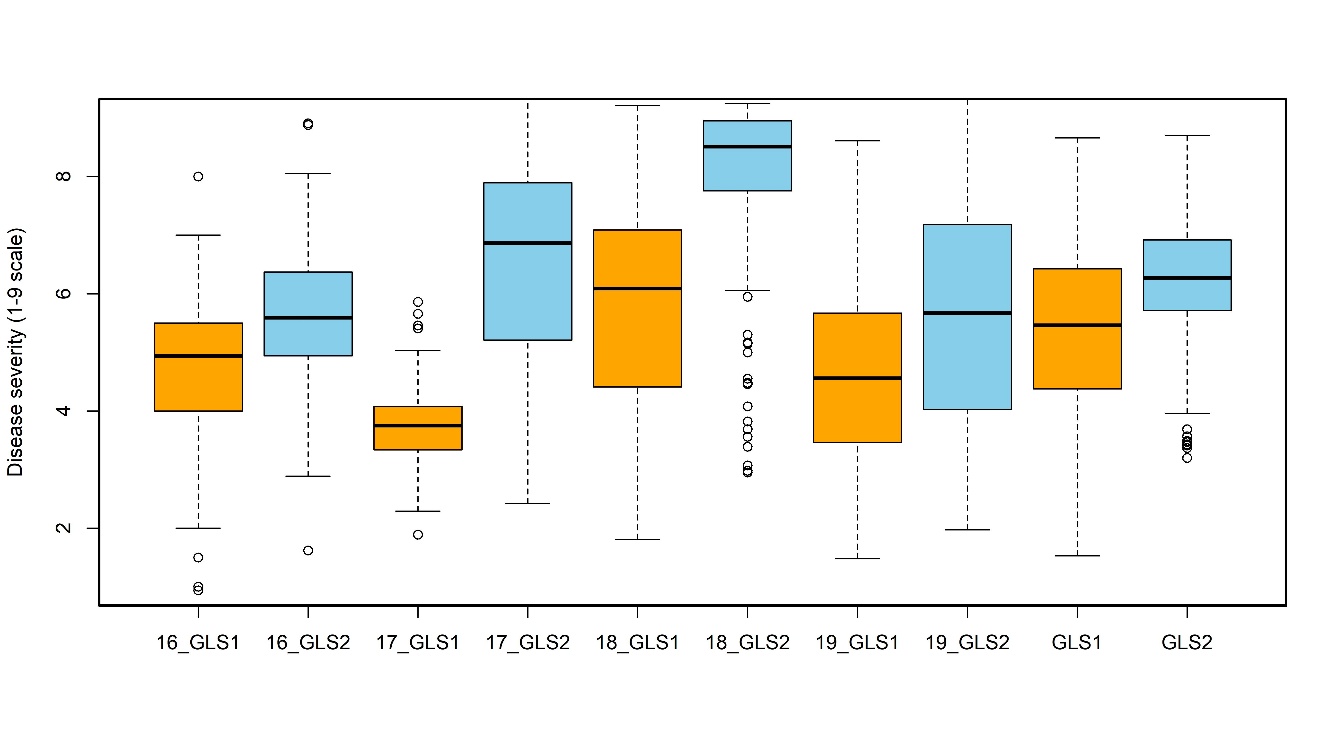


**Supplementary Figure S1.** Frequency distribution of GLS disease severity in the year 2016, 2017, 2018, 2019 and across four years. GLS1 and GLS2 = gray leaf spot disease severity data recorded at 77 and 105 days after planting, respectively.


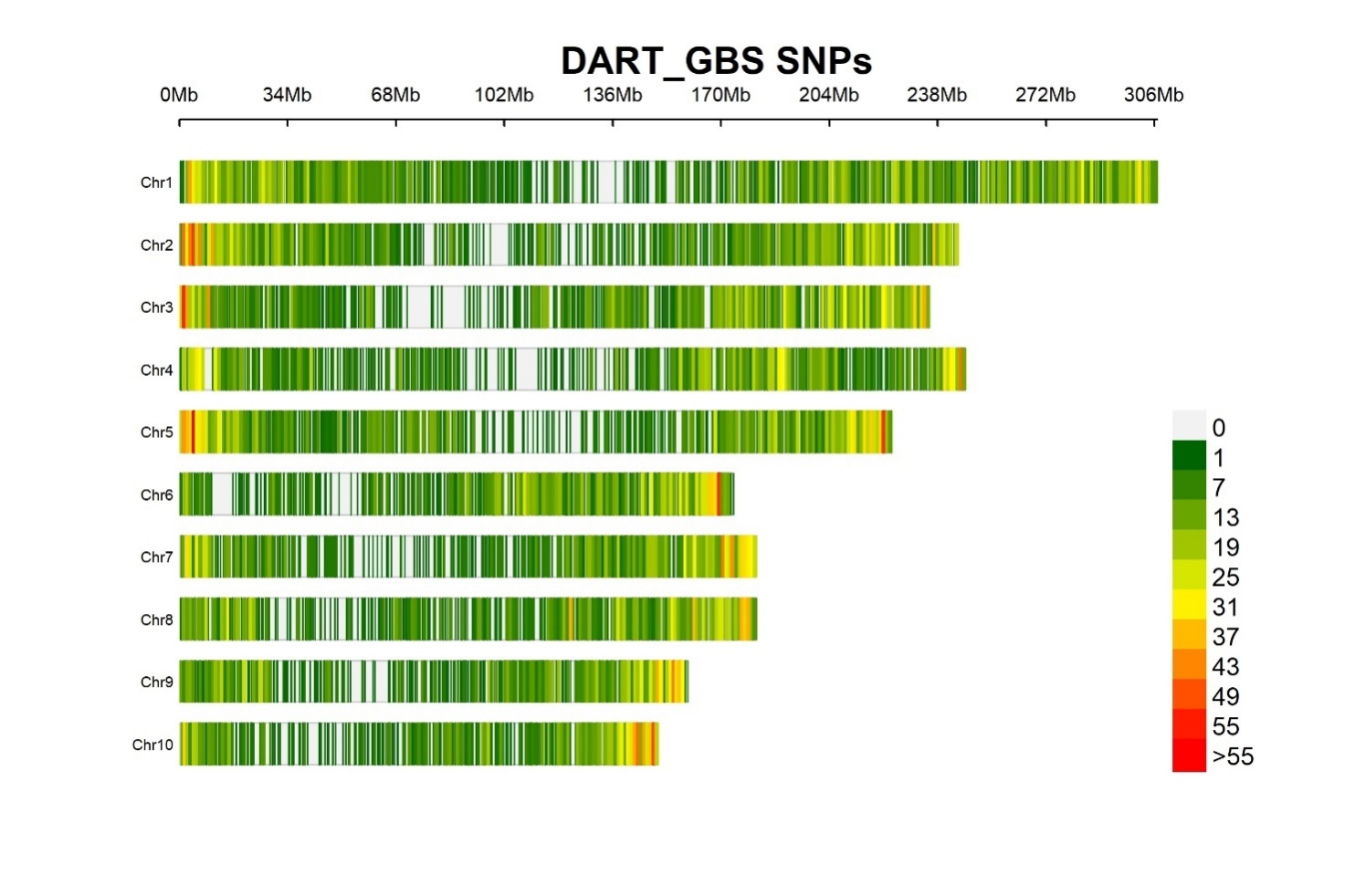


**Supplementary Figure S2.** Distribution of 19,091 hi quality DART SNPs in 10 chromosomes.


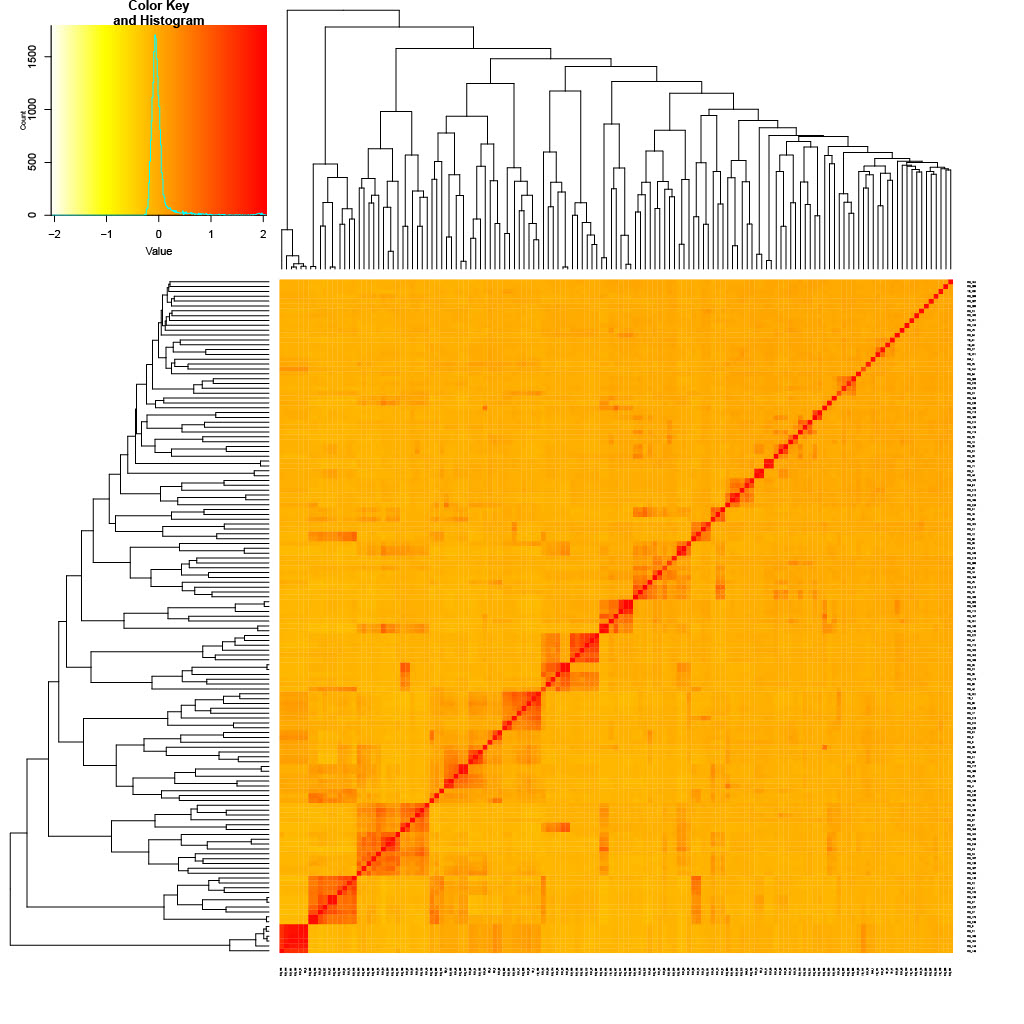


**Supplementary Figure S3.** Heat map of kinship matrix based on 19,091 DART SNPs on 140 tropical inbred lines explaining the relationship between inbred lines used in GWAS analyses, according with VanRaden algorithm. The color histogram shows the distribution of coefficients of coancestry, and the stronger red color indicates the individuals that were more related to each other.


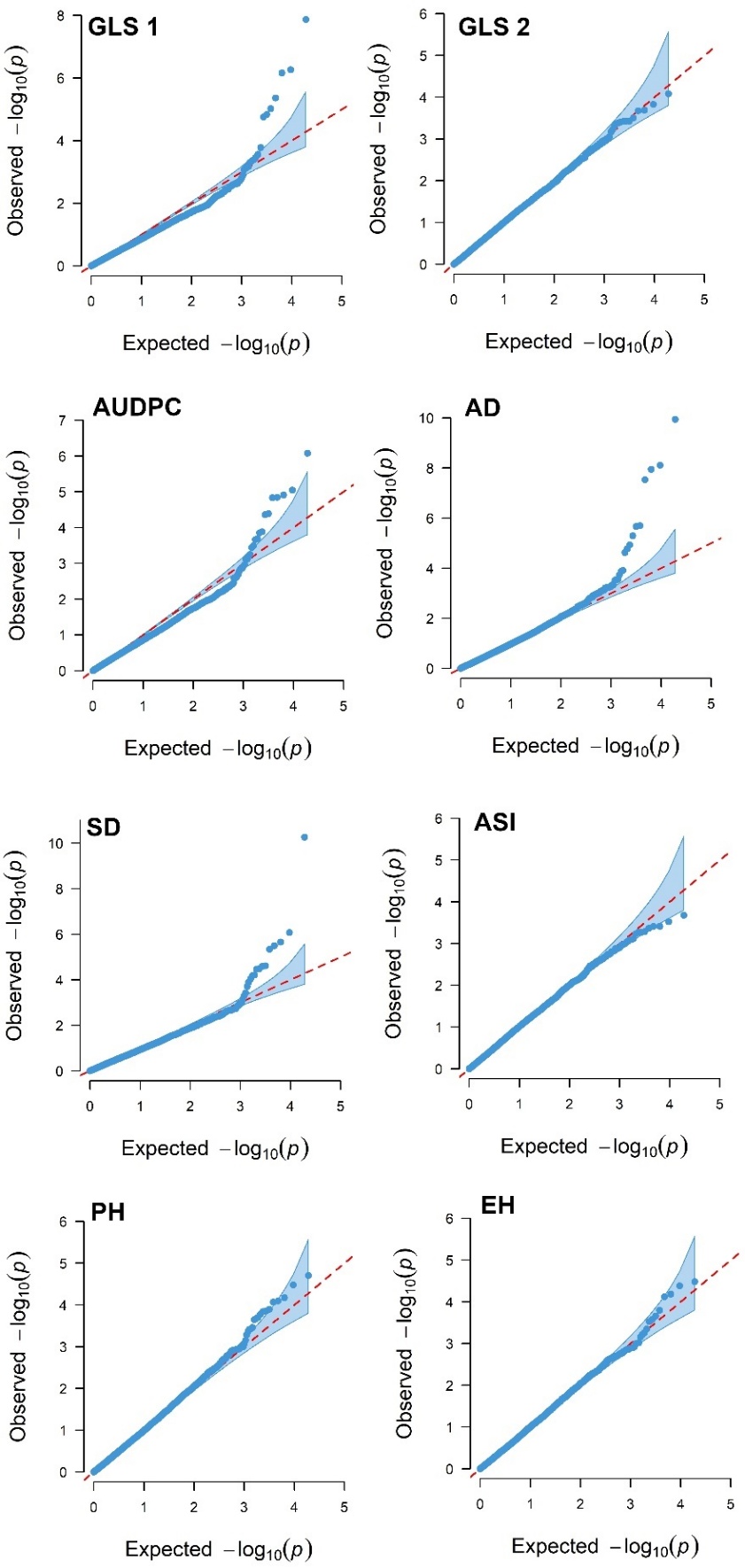


**Supplementary Figure S4.** Quantile-quantile plots of a mixed linear model for GLS resistance and other agronomic traits in the association mapping panel evaluated in four environments. The red line indicates the expected distribution under null hypothesis, and the blue line indicates the distribution from the observed association for each trait.
